# Supplementary material for: Development and Validation of a Salt Food Frequency Questionnaire (FFQ-Na) and a Discretionary Salt Questionnaire (DSQ) for the Evaluation of Salt Intake among French-Canadian Population
Source: Nutrients. 2020 Dec 30;13(1):105. doi: 10.3390/nu13010105 (PMC7824404; doi:10.3390/nu13010105)
Supplement: Supplementary file 1 [file nutrients-13-00105-s001.pdf]

Questionnaire de fréquence alimentaire pour les aliments à forte teneur en sodium (QFA-Na)

Pour chaque aliment listé ci-dessous, spécifiez tout d'abord la quantité que vous consommez habituellement en la comparant à la portion de référence indiquée (par exemple, vous avez l'habitude de consommer ½ fois, 2 fois, etc., la portion de référence). Par la suite, cochez la fréquence selon laquelle, de façon habituelle, vous avez consommé l'aliment au cours de la dernière année.

**Aliments**	**Portion de référence**	**Portion consommée**	**jamais**	**<1 fois/ mois**	**1 à 3 fois/ mois**	**1 fois/ semaine**	**2 à 4 fois / semaine**	**1 fois/ jour**	**≥2 fois/ jour**
**Charcuteries**									
1. Bacon	1 tranche		○	○	○	○	○	○	○
2. Saucisse	30 g (ou 1 saucisse à « hot dog »)		○	○	○	○	○	○	○
3. Jambon	1 tranche		○	○	○	○	○	○	○
4. Viandes froides	1 tranche		○	○	○	○	○	○	○
5. Creton	1 cuillère à table		○	○	○	○	○	○	○
6. Pepperoni/ salami/ bologne	1 tranche		○	○	○	○	○	○	○
7. Prosciutto	1 tranche		○	○	○	○	○	○	○
**Sauces**									
8. Soya ou teriyaki régulière	1 cuillère à table		○	○	○	○	○	○	○
9. Soya ou teriyaki réduite en sodium	1 cuillère à table		○	○	○	○	○	○	○
10. Barbecue	1 cuillère à table		○	○	○	○	○	○	○
11. Sauce aux tomates en conserve	1 tasse		○	○	○	○	○	○	○
12. Sauce en sachet	1 tasse préparée		○	○	○	○	○	○	○
13. Ketchup	1 cuillère à table		○	○	○	○	○	○	○
**Bouillon/ soupe/ jus**									
14. Jus de tomates/ légumes réguliers	1 petite conserve (154 ml)		○	○	○	○	○	○	○

15. Jus de tomates/ légumes réduits en sodium	1 petite conserve (154 ml)		O	O	O	O	O	O	O
16. Soupe en conserve ou en sachet	1 tasse préparée		O	O	O	O	O	O	O
17. Base de soupe en poudre	1 cuillère à thé		O	O	O	O	O	O	O
18. Concentré de bouillon liquide	1 cuillère à thé		O	O	O	O	O	O	O
**Produits céréaliers**									
19. Riz assaisonné en sachet	1 tasse préparée		O	O	O	O	O	O	O
20. Pâtes assaisonnées en sachet	1 tasse préparée		O	O	O	O	O	O	O
21. Craquelins salés	20 g (ou 6 Ritz, 7 biscuits Soda, 4 Bretons)		O	O	O	O	O	O	O
22. Craquelins réduits en sodium	20 g (ou 6 Ritz, 7 biscuits Soda, 4 Bretons)		O	O	O	O	O	O	O
23. Céréales à déjeuner	1 tasse		O	O	O	O	O	O	O
24. Pain	1 tranche		O	O	O	O	O	O	O
**Grignotines**									
25. Croustilles et/ou bretzels régulières	20 morceaux (1 tasse)		O	O	O	O	O	O	O
26. Croustilles et/ou bretzels réduites en sodium	20 morceaux (1 tasse)		O	O	O	O	O	O	O
27. Maïs soufflé régulier	1 tasse		O	O	O	O	O	O	O
28. Maïs soufflé réduit en sodium	1 tasse		O	O	O	O	O	O	O
29. Noix salées	1 poignée (¼ de tasse ou 60 ml)		O	O	O	O	O	O	O

**Fromages**									
30. Cottage	1 tasse		O	O	O	O	O	O	O
31. En brique	50 g (ou 1 tranche)		O	O	O	O	O	O	O
32. Féta	50 g (ou 1 tranche)		O	O	O	O	O	O	O
33. Fromage emballé	20 g (1 Ficello, 1 tranche Single de Kraft, 1 sachet de Tourbillon)		O	O	O	O	O	O	O
**Autres**									
34. Frites ou pommes de terre à déjeuner ou rondelles d'oignon	10 morceaux		O	O	O	O	O	O	O
35. Ailes de poulet	1 morceau		O	O	O	O	O	O	O
36. Bâtonnets de fromage panés	1 morceau		O	O	O	O	O	O	O
37. Bâtonnets de poulet/ poisson	1 morceau (10cm x 2.5cm x 1.3cm)		O	O	O	O	O	O	O
38. Thon en conserve assaisonné	1 conserve (85 g)		O	O	O	O	O	O	O
39. Cornichons et/ou olives marinés	1 cuillère à table		O	O	O	O	O	O	O
40. Légumes en conserve	1 conserve (540 ml)		O	O	O	O	O	O	O
**Mets préparés**									
41. Pâté chinois avec maïs	1 morceau (9cm x 8cm)		O	O	O	O	O	O	O
42. Pâté au poulet ou bœuf commercial	1 morceau (4 cm de diamètre)		O	O	O	O	O	O	O
43. Pain de viande	1 tranche (13 x 5 x 3cm)		O	O	O	O	O	O	O

44. Lasagne avec bœuf haché, fromage cottage et/ou mozzarella, tomates, pâte de tomates et pâtes	1 morceau (10cm x 8cm)		○	○	○	○	○	○	○
45. Pâtes avec sauce tomate ou sauce à la viande	1 tasse		○	○	○	○	○	○	○
46. Macaroni au fromage	1 tasse		○	○	○	○	○	○	○
47. Sandwich avec poulet, bacon, fromage et/ou laitue et/ou tomates et/ou mayonnaise	1 unité		○	○	○	○	○	○	○
48. Hamburger nature	1 unité		○	○	○	○	○	○	○
49. Hamburger avec fromage et/ou sauce	1 unité		○	○	○	○	○	○	○
50. Hot-dog	1 unité		○	○	○	○	○	○	○
51. Pizza avec fromage et sauce	1 pointe moyenne (ou 1/4 de 12 pouces)		○	○	○	○	○	○	○
52. Pizza garnie	1 pointe moyenne (ou 1/4 de 12 pouces)		○	○	○	○	○	○	○
53. Muffin anglais avec œuf, fromage, jambon et/ou saucisse et/ou bacon du commerce	1 unité		○	○	○	○	○	○	○

<u>Questionnaire pour la consommation du sel discrétionnaire (QSD)</u>

1. Parmi les repas que vous consommez au cours d'une semaine représentative*, combien sont …

	Déjeuner	Diner	Souper
… préparés à la maison ?			
… achetés déjà préparés ?			
… commandés du restaurant ?			
… mangés au restaurant ?			

**On entend par « représentative » ce qui décrit le mieux vos habitudes au cours de l'année.*

2. Habituellement, ajoutez-vous du sel à votre déjeuner ? (cochez)

jamais ☐ parfois ☐ souvent ☐ toujours ☐

3. Quel(s) type(s) de sel(s) utilisez-vous pour saler vos repas lors de leur préparation, de leur cuisson ou que vous ajoutez à table ? (encerclez)

1) Sel de table 2) Gros sel 3) Fleur de sel 4) Sel de céleri 5) Sel d'ail

6) Sel assaisonné 7) Sel d'oignon 8) Autre (spécifiez): _____________ 9) Aucun

4. Parmi le(s) type(s) de sel(s) que vous avez choisi(s) à la question précédente, indiquez la marque, le format et la durée d'utilisation.

Type	Marque	Format (poids en gramme)	Durée (semaine, mois, année)
Exemple :	*Windsor*	*500 grammes*	*3 mois*
**Sel de table**			
**Gros sel**			
**Fleur de sel**			

**Sel de céleri**			
**Sel d'ail**			
**Sel assaisonné**			
**Sel d'oignon**			
**Autre (spécifiez)**			

5. Quel(s) type(s) d'assaisonnement(s) salé(s) utilisez-vous pour assaisonner vos repas lors de la *préparation* ou de la *cuisson* des aliments ? (indiquez la marque, le format et la durée d'utilisation)

**Type**	**Marque**	**Format** (millilitre ou gramme)	**Durée** (semaine, mois, année)
Exemple : *sauce soya*	*VH*	*450 grammes*	*3 mois*

6. Au cours d'une semaine ou d'un mois représentatif*, quelles sont les personnes qui mangent les repas *préparés* chez vous (incluant les lunchs préparés à la maison mais consommés à l'extérieur) ?

**Membre de la famille ou autre**	**Âge**	**Déjeuner** (nombre de fois /semaine ou mois)	**Dîner** (nombre de fois /semaine ou mois)	**Souper** (nombre de fois /semaine ou mois)
Moi				
